# Neuregulin 1-ErbB module in C-bouton synapses on somatic motor neurons: molecular compartmentation and response to peripheral nerve injury

**DOI:** 10.1038/srep40155

**Published:** 2017-01-09

**Authors:** Anna Casanovas, Sara Salvany, Víctor Lahoz, Olga Tarabal, Lídia Piedrafita, Raimundo Sabater, Sara Hernández, Jordi Calderó, Josep E. Esquerda

**Affiliations:** 1 Departament de Medicina Experimental, Patologia Neuromuscular Experimental, Facultat de Medicina, Universitat de Lleida/IRBLLEIDA, Av. Rovira Roure 80, 25198 Lleida, Catalonia, Spain

## Abstract

The electric activity of lower motor neurons (MNs) appears to play a role in determining cell-vulnerability in MN diseases. MN excitability is modulated by cholinergic inputs through C-type synaptic boutons, which display an endoplasmic reticulum-related subsurface cistern (SSC) adjacent to the postsynaptic membrane. Besides cholinergic molecules, a constellation of proteins involved in different signal-transduction pathways are clustered at C-type synaptic sites (M2 muscarinic receptors, Kv2.1 potassium channels, Ca^2+^ activated K^+^ [SK] channels, and sigma-1 receptors [S1R]), but their collective functional significance so far remains unknown. We have previously suggested that neuregulin-1 (NRG1)/ErbBs-based retrograde signalling occurs at this synapse. To better understand signalling through C-boutons, we performed an analysis of the distribution of C-bouton-associated signalling proteins. We show that within SSC, S1R, Kv2.1 and NRG1 are clustered in highly specific, non-overlapping, microdomains, whereas ErbB2 and ErbB4 are present in the adjacent presynaptic compartment. This organization may define highly ordered and spatially restricted sites for different signal-transduction pathways. SSC associated proteins are disrupted in axotomised MNs together with the activation of microglia, which display a positive chemotactism to C-bouton sites. This indicates that C-bouton associated molecules are also involved in neuroinflammatory signalling in diseased MNs, emerging as new potential therapeutic targets.

Skeletal muscle contractions mediating motor behaviour involves the coordinated activity of distinct brain stem and spinal cord motor neuron (MN) populations. The firing rates of individual MNs results from the integration of a variety of excitatory and inhibitory MN synaptic inputs, which involve a range of different neurotransmitter systems. During rhythmic motor behaviour (e. g. locomotion), the highly organized pattern of MN activity is modulated by cholinergic inputs^1^. These are delivered through C-type synaptic boutons originating from spinal interneurons. C-boutons are intriguing synaptic structures that are present in α- (but not in γ-) MNs, and that were first morphologically identified over 45 years ago^2^. C-boutons are unusually large nerve terminals (3–5 μm long) which contain a high number of densely packed, round clear synaptic vesicles. In the postsynaptic region, C-terminals display a unique structure, consisting of a subsurface cistern (SSC) immediately adjacent (at 10–20 nm distance) to the MN membrane, and associated with a stack of underlying lamellae of endoplasmic reticulum (ER). There has recently been increased interest in C-boutons. After recognizing the cholinergic phenotype of C-boutons and their association with M2 muscarinic receptors^3^,^4^, other molecular aspects of these synapses, such as the expression of: voltage-gated K^+^ channels Kv2.1^5^, Ca^2+^ activated K^+^ (SK) channels^6^, vesicle-associated membrane protein 2 (VAMP-2)^3^ and sigma-1 receptors (S1Rs)^7^, have been described. The gap junction protein connexin 32 has also been localized postsynaptically at the SSC^8^,^9^. The neuronal origin of C-bouton inputs was recently identified as a small cluster of cholinergic interneurons (VOc interneurons) located near the central canal of the spinal cord, which modulate MN activity during locomotor behaviour^1^,^10^. Metabotropic M2 transmission in C-type synapses may inhibit Ca^2+^ dependent K^+^ channels, reducing the spike afterhyperpolarization, instead of after hyperpolarization and enhancing MN firing frequency through a local intermediary effect of [Ca^2+^]_in_^1^,^10^.

The neuregulin-1 (NRG1) family includes more than 15 membrane-associated and secreted growth factors generated by alternative RNA splicing that interact with ErbB receptor tyrosine kinases. All NRG1 isoforms share an epidermal growth factor (EGF)-like signalling domain, and control many aspects of development. In the nervous system, the most abundant forms of NRG1 are types I and III. These, have been detected in MNs, dorsal root ganglion (DRG) neurons and, in particular in glia. NRG1 is involved in glia-axon interactions (myelination), the development of motor endplates, neuronal migration, synaptogenesis and plasticity in the CNS^11^,^12^,^13^,^14^. NRG1 is also involved in psychiatric disorders, including schizophrenia^15^,^16^. In a study examining the expression of NRG1 in phrenic spinal cord MNs, it was reported that this trophic protein is expressed in nearly all cholinergic nerve terminals^17^; however, its precise subcellular localization was not addressed by these investigators. We have recently examined this issue and found that NRG1 is associated with C-boutons, concentrated in postsynaptic sites within the ER at the SSC^18^. We have also demonstrated that ErbB2 and ErbB4 receptors are present in the presynaptic compartment, suggesting that NRG1 acts as a retrograde signalling molecule in C-type synapses. C-boutons are scarcely investigated structures^19^, which have been reported to suffer plastic changes in sick MNs after spinal cord injury^20^,^21^, peripheral nerve lesion^22^, and amyotrophic lateral sclerosis (ALS)^18^,^23^,^24^,^25^,^26^,^27^,^28^,^29^. In addition, mutations in S1R are considered risk factors for familial ALS, and abnormal accumulations of this protein in C-terminals have also been observed in patients with sporadic ALS^30^,^31^,^32^. Altered SSCs also appear to be present in ALS-linked mutations of vesicle-associated membrane protein-associated protein B (VAPB, ALS8), which is abnormally targeted to C boutons^33^.

The relevance of the NRG1/ErbB pathway in MN diseases has been further underlined by the discovery of a new type of familial ALS caused by a loss of function mutation of ErbB4^34^, and by the reported neuroprotective effects of NRG1 supplementation^35^ and/or altered NRG1 expression^36^ in ALS mice. MNs in oculomotor nuclei, which are spared in ALS, lack both C-boutons and SSC-associated NRG1^4^,^18^.

Thus, at C-type synaptic sites, in addition to the presence of cholinergic molecules, a constellation of proteins involved in different signal-transduction pathways are specifically clustered, although their functional significance remains largely unknown. In order to better understand synaptic signalling though C-boutons, we undertook a structural analysis of the spatial organization of distinct C-bouton-associated signalling proteins, and especially the NRG1-ErbBs module, in different MN populations. We also examined changes in NRG1-ErbB expression during development and following peripheral nerve injury.

## Results

### Ultrastructure of C-bouton synapse in mouse spinal cord MNs

C-boutons can be morphologically distinguished from other afferent synaptic terminals contacting MN soma or dendrites in the spinal cord ventral horn. The C-bouton presynaptic element contains spherical and electron lucent synaptic vesicles, some endosome-like bodies and mitochondria. In the postsynaptic compartment, and in close apposition with the postsynaptic membrane, the ER-related SSC is the main structural hallmark of C-boutons. The distance between the SSC and postsynaptic membrane is about 10–15 nm. C-boutons with large SSCs were also observed in MNs of newborn (P1) animals (Supplementary Fig. 1a–d). Our observations are consistent with the classical description of C-boutons in cat spinal cord^2^.

### The NRG1-ErbB module is found in C-boutons and is differentially compartmentalised with other signalling proteins

**Figure 1:**
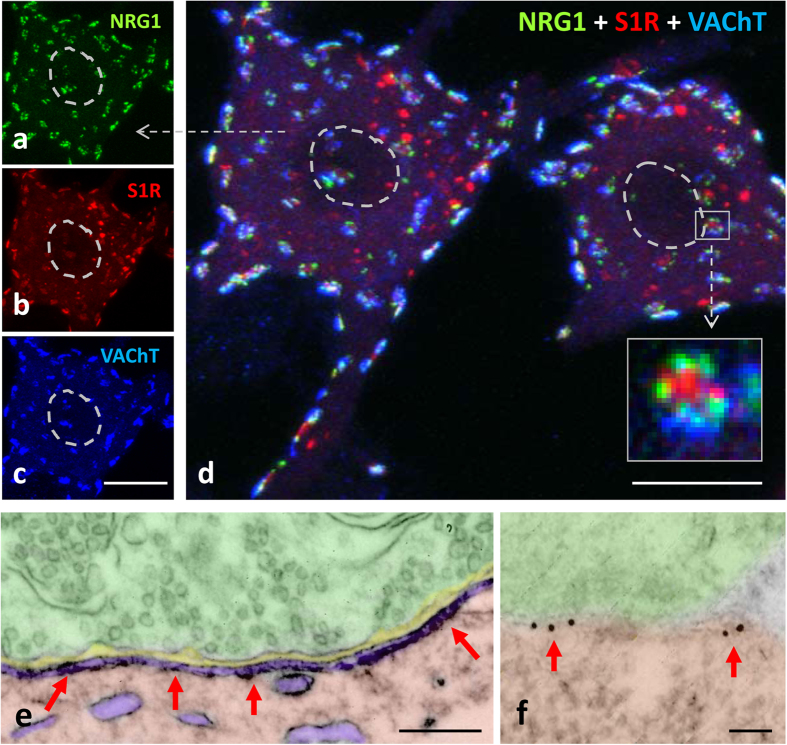
NRG1 protein is associated with the C-bouton proteins VAChT and S1R, and localized in SSC at postsynaptic region. **(a–d)** Squashed MNs triple immunolabelled for NRG1 (green), S1R (red) and VAChT (blue). The three molecules colocalise at C-boutons, but S1R is also present as non-synaptically-associated particles. The nuclei are delimited by a dashed line. Individual RGB channels of the indicated MN (arrow) are shown in **(a-c).** When individual C-boutons are observed at high magnification (square delimited in **(d)**), sites containing S1R are segregated from NRG1-positive patches, and delimited by presynaptic VAChT. **(e** and **f)** NRG1 accumulates in the SSC (arrows) as shown in spinal cord MNs after ultrastructural immulolabelling by a pre-embedding **(e)** or post-embedding immunogold **(f)** procedure. Scale bars: in **(c)** = 10 μm; **(d)** = 20 μm; in **(e)** = 250 nm; in **(f)** = 50 nm.

**Figure 2:**
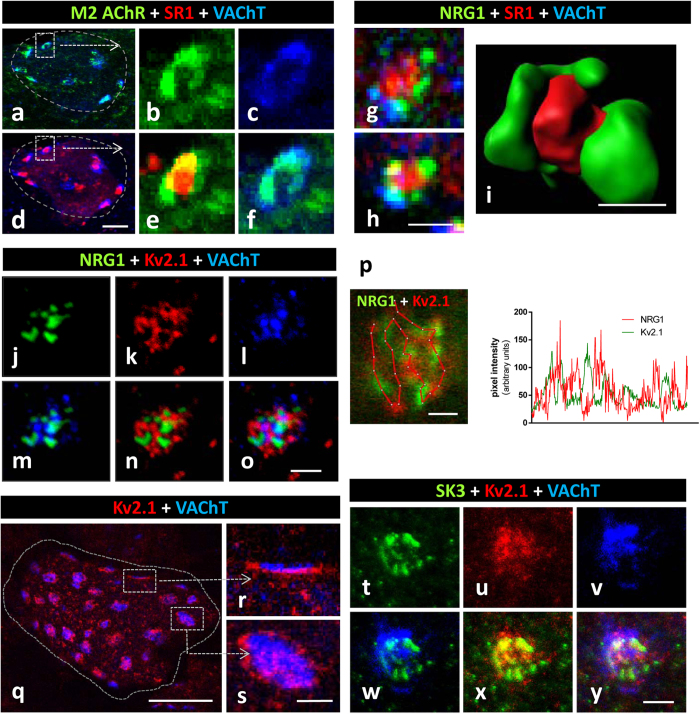
C-bouton proteins NRG1, S1R, Kv2.1 and SK3 are differentially clustered at the postsynaptic region. (**a–f)** MN somata (encircled in **(a)** and **(d)**) showing C-bouton synapses immunostained for simultaneous visualisation of M2 AChR (green), SR1 (red) and VAChT (blue); an enlargement of the square delimited C-bouton is shown in **(b,c,e,f)** in either separate or combined channels; note the similar distribution of the M2 AChR and VAChT sites; this contrasts with the differential localisation of M2 AChR and SR1; two channel merged images are shown in **(a,d,e** and **f)**. **(g,h)** Sites containing S1R locate in a central region surrounded by NRG1-positive patches, separately delimited from presynaptic VAChT. **(h)** Volume rendering of a C-bouton site showing the differential distribution of NRG1 (green) and S1R (red) is depicted. **(j–o)** Clusters of NRG1 (green) and Kv2.1 (red) display a complementary distribution within the VAChT-positive (blue) C-bouton; two and three channel merged images are shown in **(m,n)** and **(o)**, respectively. **(p)** A detail of a C-bouton, double labelled for NRG1 (green) and Kv2.1 (red), is subjected to pixel profile analysis along the depicted line; note the absence of colocalisation between the two interdigitating signals. **(q–s)** Surface of squashed MN cell body (delimited by a dotted line) displaying the distribution of clustered Kv2.1 (red) potassium channels in VAChT-immunolabelled C-boutons (blue) **(q)**; the lateral and orthogonal projections of enlarged C-boutons are shown in **(r)** and **(s)**, respectively; the postsynaptic localisation of Kv2.1 channels is shown in front of a VAChT-positive terminal in **(r)**; the belt like arrangement of Kv2.1 potassium channels around the VAChT-labelled C-bouton can be seen in **(s)**. **(t–y)** A triple immunolabelled C-bouton showing the differential distribution of SK3 Ca^2+^-dependent K^+^ (green) with respect to Kv2.1 K^+^ (red) channels in a VAChT-delimited (blue) synaptic region; two and three channel merged images are shown in **(w,x)**, and **(y)** respectively. Scale bars: in **(d)** = 10 μm (also applicable to **(a)**); in **(h)** (valid for **(g)**), **(i)**, **(o)**, **(p)**, **(s)** (valid for **(r)**) and **(y)** = 1 μm; in **(q)** = 20 μm.

Using several specific antibodies we analysed the precise distribution of several proteins that are known to be clustered at the C-type synapse. C-bouton sites were identified by labelling presynaptic terminals with vesicular acetylcholine transporter (VAChT, Figs 1 and 2). In close association with VAChT-positive spots, we confirmed the presence of NRG1, M2-type muscarinic acetylcholine receptors (M2 AChRs), S1Rs, the Kv2.1 delayed rectifier K^+^ channel (Kv2.1), and the postsynaptic SK channel SK3. All of these were present in oval patches distributed throughout the soma and in proximal dendrites of large spinal cord MNs, representing the postsynaptic compartment of C-bouton (Fig. 1a–d). In agreement with our previous work^18^, non-cholinergic (i.e. glutamatergic, serotoninergic and GABAergic) afferent synapses on MNs did not express the particular assembly of postsynaptic molecules seen in cholinergic terminals (not shown). In addition, ultrastructural immunolabelling (Fig. 1e,f) followed by the stereological analysis^37^ of gold nanoparticles distribution confirmed that, within C-bouton, NRG1 was accumulated postsynaptically in the SSC (for total Chi-square = 19,337.8 and df = 4, *p* < 0.0001, there is a preferential labelling in SSC, n = 20 C-bouton synapses; see Fig. 1f).

**Figure 3:**
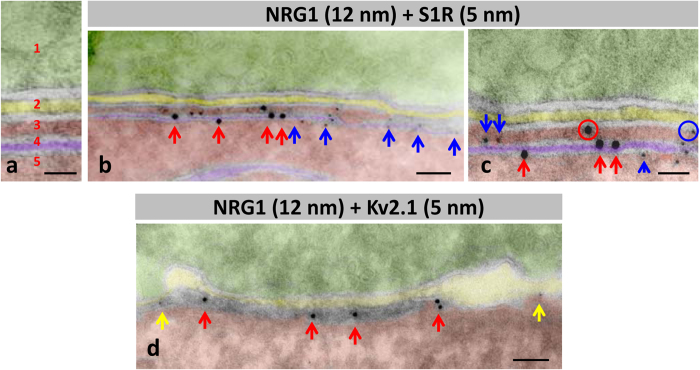
Immunolabelling using nanogold particles of ultrathin cryosection for the ultrastructural localisation of NRG1, S1R and Kv2.1 channels at C-boutons. To facilitate the localisation of C-boutons, the analysis was performed in hypoglossal MNs. A detail of the organization of compartments at C-bouton synapses in negatively stained cryosections is depicted in (**a**) 1 = presynaptic (green), 2 = intersynaptic extracellular space (yellow), 3 = postsynaptic cytoplasmic compartment lodged between postsynaptic membrane and subsynaptic cistern (SSC, red), 4 = SSC (violet), and 5 = MN cytoplasm (red). The same colour code is used in (**b–d**). (**b,c**) Double immunolabelling for NRG1 and SR1; NRG1 (red arrows) is mainly associated with SSC forming a cluster segregated from SR1 (blue arrows). (**c**) A minor proportion of gold particles (encircled) are located at the postsynaptic membrane. (**d**) nanogold particles labelling Kv2.1 are located on the periphery (yellow arrows) of C-bouton enriched with a NRG1 cluster (red arrows). Scale bar: in (**a,c**) = 25 nm; in (**b,d**) = 50 nm.

C-bouton-associated protein molecules were assembled in a highly ordered manner and segregated into specific microdomains. This was clearly shown after confocal imaging of multi-labelled C-boutons using the highest resolution settings. Our results complement those reported by Deardorff *et al*.^19^. Postsynaptic sites enriched in M2 cholinergic receptors were precisely aligned with the strongest signal for VAChT, indicating the areas with the maximum concentration of cholinergic vesicles at the presynaptic terminal (Fig. 2a–f). The areas most enriched in NRG1 were intercalated with microdomains displaying the maximal intensity for Kv2.1 or S1R immunolabelling (Fig. 2g–p). In orthogonal projections of C-boutons, the S1R signal was usually detected in the form of a central saccule-like structure surrounded by smaller NRG1-containing spots (Fig. 2g–i). In addition, in some MNs, Kv2.1 immunoreactivity formed an additional bell-like line, externally surrounding the whole C-bouton area (Fig. 2q–s). This is in agreement with observations made in hippocampal neurons^38^,^39^. The segregated localization of NRG1 and Kv2.1 in C-type synapses was further assessed after double immunolabelling. This analysis suggested that Kv2.1 subunits were located both in the postsynaptic membrane and in the SSC, although they were clustered in separate domains than NRG1 (Fig. 2p). SK3 channel protein was also clustered in C-boutons from some, but not all, MNs; these, probably correspond to MNs innervating slow-twitch muscles^6^. In our samples, SK3 and Kv2.1 potassium channels appear to be localized in separate domains (Fig. 2t–y). All these results indicate that the postsynaptic machinery at C-boutons contains a unique mosaic of proteins that are spatially arranged in highly ordered, interdigitated, non-overlapping, microclusters. Using double immunogold labelling, the regions within the SSC enriched in NRG1 and S1R were clearly segregated; most of the particles were located at the SSC membrane. A minor proportion (9.7 ± 2.59%, n = 14 synapses) of nanogold particles were seen associated with the postsynaptic membrane, suggesting that NRG1 may have been translocated from the SSC to the postsynaptic membrane (Fig. 3a–c). Double immunogold labelling was also applied to identify NRG1 in conjunction with Kv2.1; results confirmed that both proteins reside postsynaptically in separate regions, with Kv2.1 located at the periphery of clustered NRG1 (Fig. 3d). These data extend previous observations on the ultrastructural localization of Kv2.1 channels^5^,^38^. To improve C-bouton sampling in ultrathin cryosections, all these double labelling experiments were performed in hypoglossal MNs, as these are more densely packed than ventral horn MNs. We have already described that both morphological and immunocytochemical properties of C-boutons in hypoglossal MNs are virtually identical to those in spinal cord^18^.

**Figure 4:**
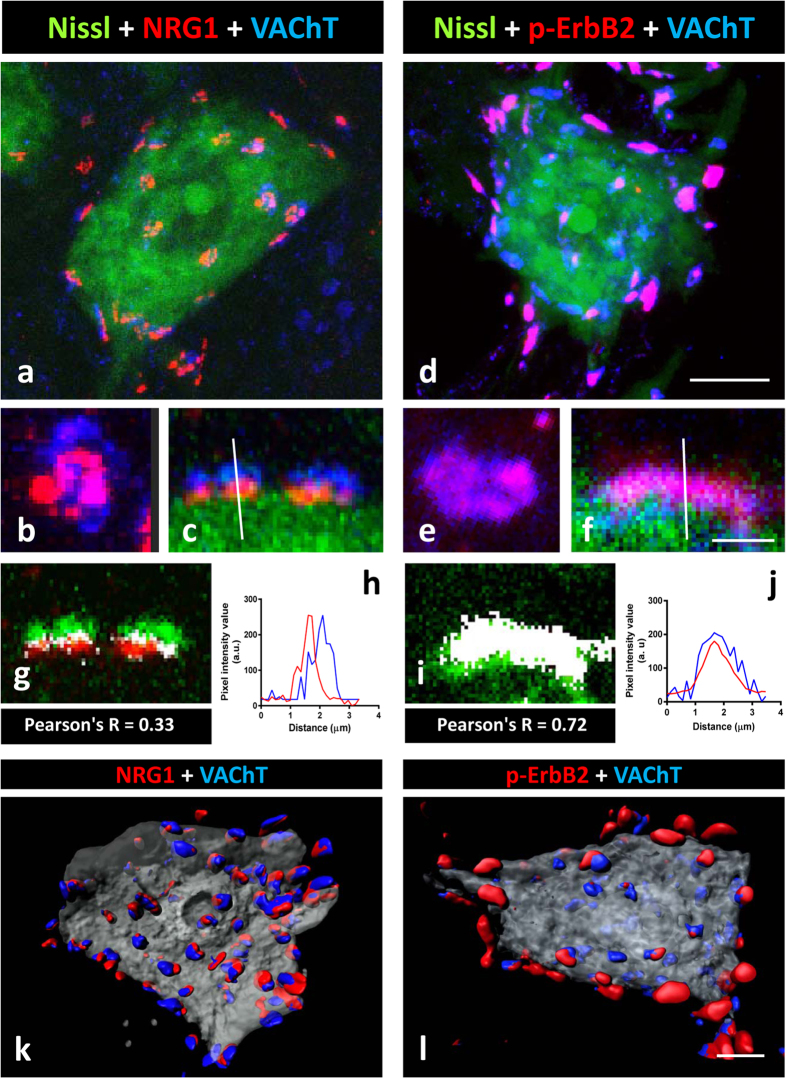
C-bouton type synapses contain the NRG1/ErbB signalling module. **(a**,**b,c)** NRG1 (red) is concentrated at the C-bouton postsynaptic site adjacent to VAChT (blue) positive cholinergic terminals. **(d**,**e,f)** show that p-ErbB2 (red) is present in the presynaptic site of many, but not all, cholinergic terminals labelled with VAChT antibody (blue). In **(a,c,d,f)** MN somata are visualised with fluorescent Nissl staining (green). In **(b)** and **(c)**, orthogonal and lateral projections, respectively, of a C-bouton immunolabelled for NRG1 and VAChT are shown; the dissociation of the two signals is evidenced after colocalisation **(g)** and pixel profile analysis **(i)**. In **(e)** and **(f)** orthogonal and lateral projections, respectively, of a C-bouton immunolabelled for p-ErbB2 and VAChT are also shown; the overlapping of two signals is evidenced after a colocalisation **(h)** and pixel profile analysis **(j)**. Colocalised pixels are displayed in white in **(g)** and **(i)**; the low numbers of white pixels in **(g)** corresponds to the boundary between pre- and post-synaptic compartments, which are overlapped due to mechanical folding and compression inherent to tissue processing. **(k,l)** represents the volume rendering of serial optical sections (0.5-μm thick) from a MN showing the distribution of NRG1 (red in **(k)**) and p-ErbB2 (red in **(l)**), and VAChT (blue) co-labelled C-boutons. Scale bars: in **(d)** = 10 μm (also applicable to **(a)**); in **(f)** = 3 μm (also applicable to **(b, c, e)**); in **(l)** = 5 μm (valid for **(k)**).

By using several anti-ErbB2 or ErbB4 antibodies, positive immunolabelling in the presynaptic compartment of C-boutons was unambiguously observed in contrast to the postsynaptic localisation of NRG1 (Fig. 4a–c). Although this immunoreaction was sometimes faint and imprecise, it became more prominent when phosphospecific antibodies were used and, in particular, after performing tyramide signal amplification (TSA) (Fig. 4d–f, and Supplementary Fig. 2). The immunostaining obtained with the anti-phosphorylated ErbB2 or ErbB4 antibodies overlapped the presynaptic VAChT positivity in most, although not all, C-boutons (Fig. 4d). However, unphosphorylated ErbB2 and ErbB3 were not detected in association with VAChT-positive puncta. The plot profile and co-localization analysis showed a high degree of coincidence between phospho-ErbB2 and VAChT signals (Pearson’s R = 0.72, Fig. 4i,j). By contrast, the same analysis confined NRG1 to the postsynaptic compartment, and as being clearly distinct from the VAChT signal (Pearson’s R = 0.33, Fig. 4g,h). The intensity of ErbB immunoreactivity varied among the individual cholinergic terminals, ranging from negative or very weak to intense. In addition to the synaptic immunolabelling, ErbB2 and ErbB4 were also detected in MN cell bodies and neuropil, particularly when TSA was used. The association between either NRG1 or p-ErbB2 with VAChT immunolabelled C-boutons can be further appreciated after volume rendering imaging (Fig. 4k,l).

**Figure 5:**
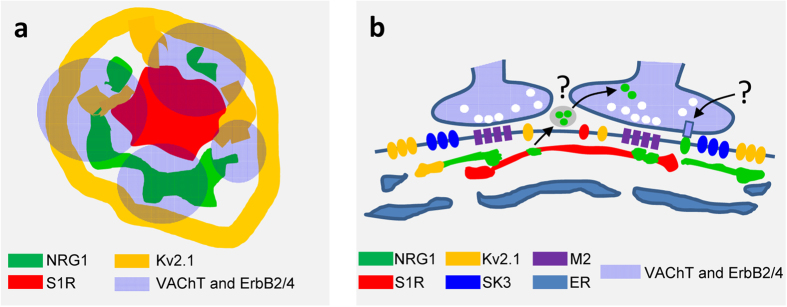
Model showing the possible distribution of some proteins specifically concentrated at C-type synapses. The synapse is displayed in orthogonal **(a)** and lateral **(b)** views. The different sub-compartments in which proteins accumulate are depicted according to the indicated colour code. Since the mechanism by which postsynaptic NRG1 and presynaptic ErbBs interact is unknown, two possible routes are indicated with question marks: one mediated by exosomes, and the other involving a juxtacrine interaction.

This overall spatial distribution of C-bouton-associated proteins is depicted in Fig. 5(a,b), based on a model that we proposed after examining 3D reconstructions of multi-labelled C-bouton synapses.

The total number of C-boutons per MN soma was calculated from 3D reconstructed squash preparations of thick (200 μm) spinal cord slices from adult mice. After analysing 14 MNs, we found an average of 3.11 ± 0.30 (in 100 μm^2^ of MN soma surface; C-type synapses were visualized by VAChT immunostaining). Similar numbers were found after evaluating clustered NRG1-positive particles (3.60 ± 0.29/100 μm^2^ of MN soma surface) and synaptically-associated SR1-positive spots (2.92 ± 0.28/100 μm^2^). The average size of the synaptic area containing the distinct C-bouton markers was as follows: VAChT = 6.87 ± 0.25, n = 184; NRG1 = 5.41 ± 0.20 n = 203; SR1 = 4.30 ± 0.20, n = 153 (all in μm^2^; with n corresponding to the number of C-bouton examined). We further determined whether these morphometric parameters differed when the analysis was specifically performed in MN subtypes innervating fast or slow muscles. MN pools were identified after retrograde tracing by injecting fluorescent cholera toxin B into either tibialis anterior (TA) or soleus muscles (Supplementary Fig. 3). The results indicated that NRG1-positive puncta were more abundant, although smaller, in fast type TA-innervating MNs than in slow type MNs contacting the soleus muscle.

### Development of C-bouton-associated NRG1

**Figure 6:**
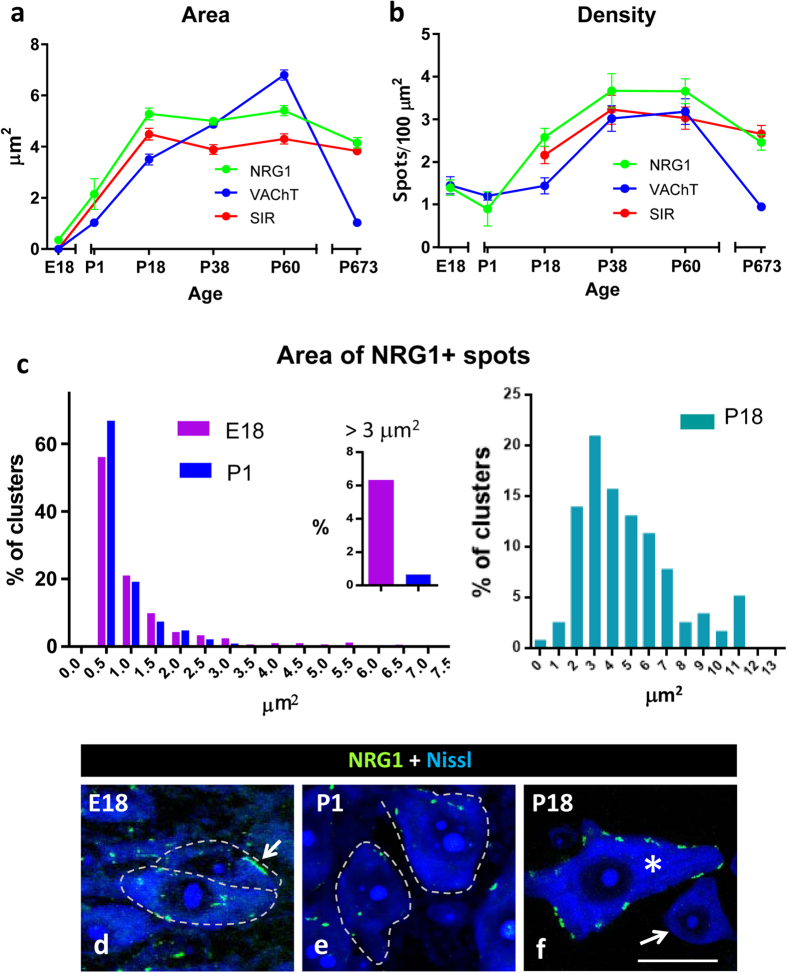
Age-related changes in the morphometrical parameters of the C-bouton associated proteins NRG1, VAChT and S1R. The area and density of the immunolabelled protein profiles are shown in **(a)** and **(b)**, respectively. **(c)** Relative frequency histogram of NRG1 clusters for late embryonic (E18), newborn (P1) and P18 MNs; note that the large (>3 μm^2^) NRG1 clusters seen on E18 animals are substantially reduced by P1. **(d–f)** Representative images of MN somata used for quantification in **(c)**; arrow in **(d)** indicates a large cluster of NRG1 on an E18 MN; MN somata are delimited by dashed lines; note that at P18 **(f)**, C-bouton-associated clusters of NRG1 were only seen on large MNs (*), but not on smaller cell bodies corresponding to interneurons (arrow). In all the graphs, the data are shown as mean ± SEM. One-way analysis of variance (Bonferroni’s post-hoc test) was used for statistical analysis. Scale bar in **(f)** 20 μm (valid for **(d,e)**).

Developmental changes in C-bouton associated proteins such, as Kv2.1 and M2 AChRs occur postnatally, together with the functional maturation of the motor system^40^. We analysed the developmental expression of NRG1 in the spinal cord in relation to the other C-bouton associated molecules, such as VAChT and SR1 (Fig. 6a–f). Small (<1 μm^2^), but abundant, NRG1 positive discrete puncta were detected prenatally at E18 (Fig. 6c,d). Many of these particles did not show any association with VAChT-positive presynaptic terminals. In addition, at this stage, large patches of NRG1 (>3 μm^2^) were often seen adjacent to the MN surface, although they were not related to VAChT-labelled nerve terminals. These NRG1 clusters, underwent a conspicuous remodelling becoming virtually absent from P1 onwards (Fig. 6c,e). During the postnatal period, the size of the NRG1 clusters increased progressively, reaching a plateau at P18 (Fig. 6a,b,f). At this age, virtually all of the NRG1 clusters were associated with presynaptic cholinergic terminals. In agreement with Mavlyutov *et al*.^41^, C-bouton-associated S1R immunoreactivity began only after the second week of postnatal development (Fig. 6a,b). In the spinal cord MNs of aged mice, the density and size of the C-bouton presynaptic terminals were markedly reduced, whereas this did not occur for the postsynaptic proteins NRG1 and S1R.

**Figure 7:**
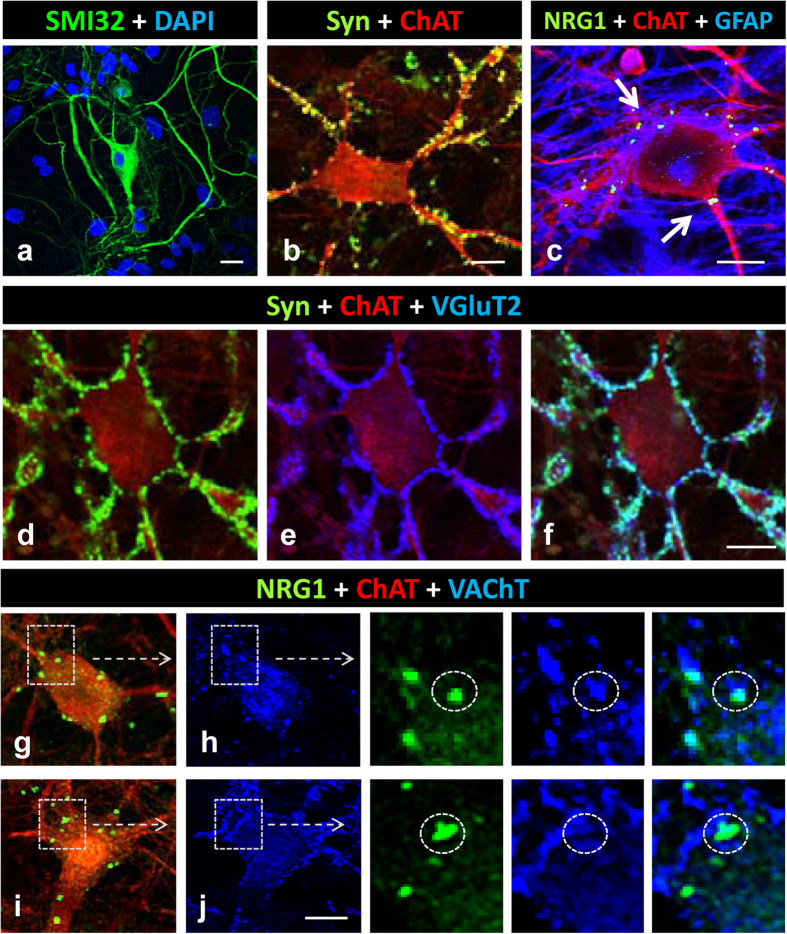
Formation of synaptic afferents in long-term cultures of spinal cord neurons. MNs are identified with SMI32 or ChAT immunostaining. **(a)** A representative highly branched SMI32 (green) positive MN. **(b)** A ChAT positive (red) MN showing multiple afferent synaptic boutons detected by synaptophysin (Syn, green) labelling. **(c)** A ChAT positive (red) MN displaying NRG1 clusters (green) which, in some cases, are in contact with GFAP positive (blue) astroglial profiles (arrows). **(d–f)** A ChAT positive (red) MN with multiple Syn positive (green) synaptic contacts in which many contain VGluT2 (blue). **(g,h)** A ChAT positive MN (red) showing VAChT positive (blue) cholinergic afferent boutons in close association with NRG1 (green) positive clusters, as detailed (encircled) in the enlarged region delimited in **(h)**. **(i,j)** A ChAT positive MN (red) showing NRG1 (green) positive clusters which are not associated with VAChT (blue) positive puncta, as shown (encircled) in the enlarged region delimited in **(j)**. Scale bars: in **(a** and **b)** = 20 μm; in **(c)** = 10 μm; in **(f)** = 20 μm (valid for **(d,e)**); in **(j)** = 20 μm (valid for **(g,h,i)**).

In order to analyse NRG1 and C-bouton synapse formation in an *in vitro* model, we performed long-term cultures of dissociated spinal cord neurons. We assumed that the presence of a mixed neuronal population would be necessary to develop C-bouton-like cholinergic afferents and that the presence of glial cells would provide trophic support for promoting long-term MN survival and differentiation. In these cultures, highly branched and large MNs were identified after immunostaining with either SMI32 or anti-ChAT antibodies following 14–28 days *in vitro* (Fig. 7a,b). These MNs exhibited neurite outgrowth over long distances and formed an interconnected network with abundant neuritic-neuritic and neuritic-somatic junctures, which displayed the synaptic marker synaptophysin (Syn) and appeared distributed in discrete puncta. Many of these were glutamatergic boutons because they also contained VGluT1 or VGluT2 (Fig. 7d–f). Very few MNs showed VAChT-positive axo-somatic or axo-dendritic synaptic contacts adjacent to spots containing NRG1 (Fig. 7g,h). By contrast, a variable number of spots with a prominent NRG1 signal was observed in many MNs without any association with the presynaptic nerve terminal markers Syn and VAChT (Fig. 7i–j). These non-synaptic associated NRG1 patches were often in close proximity to GFAP-positive astroglial processes (Fig. 7c). It is interesting to note that somato-dendritic NRG2 puncta have been described in association with astroglia in cultured hippocampal neurons^39^. Overall, this *in vitro* model recapitulates some of the early events leading to C-bouton formation *in vivo,* but it is only able to generate afferent cholinergic synapses on a minor proportion of MNs.

### Disruption of postsynaptically-clustered NRG1 in axotomised MNs together with microglial activation

**Figure 8:**
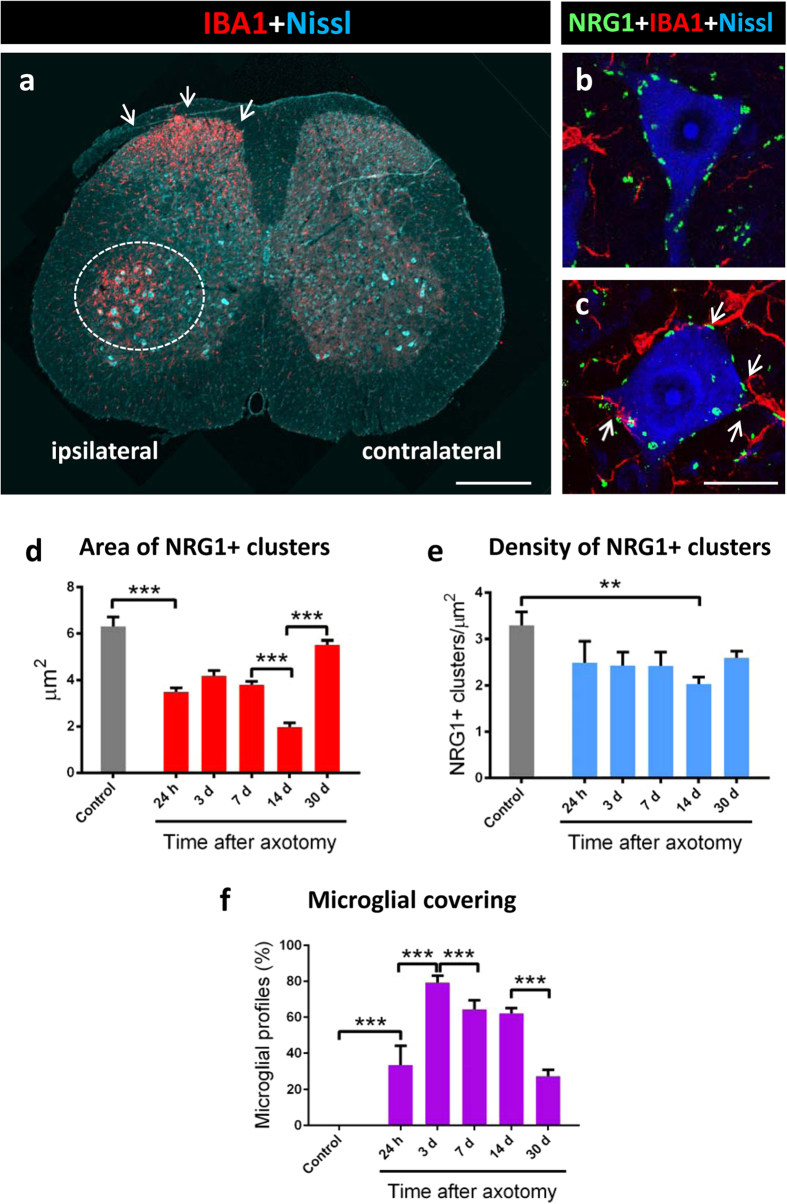
C-bouton-associated NRG1 clusters are disrupted after peripheral nerve transection together with microglial activation. **(a)** A lumbar spinal cord transverse section 7 days after unilateral sciatic nerve transection; note the microglial reaction (IBA1, red) around the ventral horn MNs (encircled) and in the dorsal horn (arrows); the section was Nissl-counterstained (blue). **(b,c)** Detail of spinal MNs corresponding to the contralateral **(b)** and ipsilateral **(c)** ventral horns following nerve axotomy stained for NRG1 (green), IBA1 (red) and Nissl (blue); microglial cells are recruited around axotomised MNs **(c)**, in which NRG1 clusters are in the process of fragmentation. Note the tendency of microglial profiles to contact altered NRG1 clusters. The time course of changes in the morphometrical parameters of NRG1 clusters after sciatic nerve axotomy are shown in **(d,e)**. Changes in the perisomatic-microglial covering of MNs are measured at the same time points **(f)**. In all the graphs, data are shown as mean ± SEM. **p < 0.01, ***p < 0.001, one-way analysis of variance (Bonferroni’s post-hoc test); n = 27–113 **(d)**; n = 7–15 **(e)** and n = 10–18 **(f).** Scale bar: in **(a)** = 250 μm; in **(c)** = 20 μm (valid for **(b)**).

**Figure 9:**
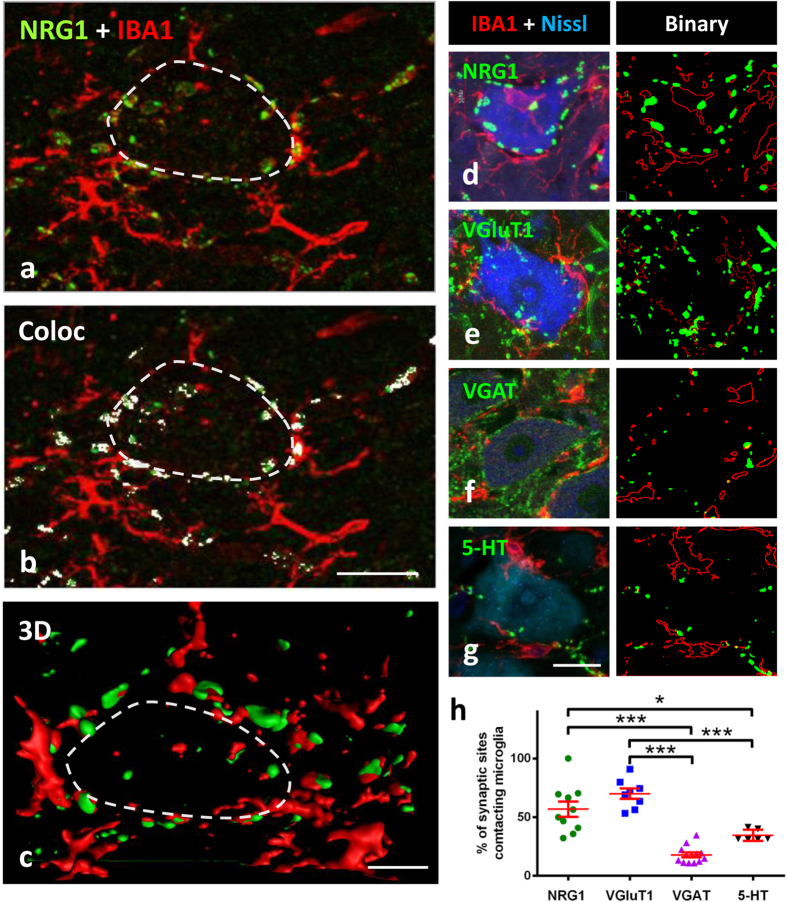
Recruitment of microglial cells to axotomised MNs exhibits apparent positive chemoattraction to C-bouton sites. **(a)** IBA1 positive microglial profiles (red) near the soma of a 24 h axotomised MN show a high number of contacts with NRG1 positive C-bouton sites (green). **(b)** After colocalisation analysis, NRG1 clusters enwrapped by microglia are displayed in white. **(c)** 3D volume rendering showing the relationship between microglial processes and NRG1 clusters in the MN displayed in **(a)**; the limit of MN soma is delineated (dashed lines). **(d–g)** The spatial relationship between microglial profiles and different afferent boutons on 7-day axotomised MNs is analysed after immunostaining for IBA1 (red) and the indicated synaptic proteins (green); MN somata were identified by Nissl staining (blue). The green and red signals were binarised for analysis as shown in each adjacent panel. **(h)** Graph showing that microglial profiles exhibit a preference to contact NRG1-labelled C-boutons and/or VGluT1-containing synapses, rather than to VGAT-immunolabelled GABAergic or serotoninergic (5-HT) synapses; plotted data are also shown as mean ± SEM; *p < 0.05, ***p < 0.001, one-way analysis of variance (Bonferroni’s post-hoc test). Scale bars: in **(b)** = 20 μm (valid for **(a)**); in **(c)** = 5 μm; in **(g)** = 20 μm (valid for **(d,e,f)**).

The retrograde response of neurons to peripheral nerve interruption is associated with prominent changes in ER organisation^42^, in the plasticity of synaptic inputs combined with the activation of perineuronal glial cells^43^,^44^, and increased excitability^22^. As these changes may be related to altered C-bouton function^22^, we analysed the impact of sciatic nerve transection on postsynaptic NRG1. In spinal cord sections, the pool of axotomised MNs was readily identifiable after IBA1 immunostaining due to the notable and early recruitment of microglia around lesioned MN somata (Fig. 8a). As shown in Fig. 8b–f, a progressive and transient disintegration of NRG1 clusters occurred in axotomised MNs associated with perisomatic microglial activation starting 24 h after nerve injury. Although the reduction in size of NRG1 immunoreactive profiles reached its maximum 14 days following axotomy, the density of individual clusters was only slightly changed. By 30 days post-lesion, the size of the NRG1 profiles found in basal conditions showed a recovery, reaching similar values to controls. Correlative ultrastructural alterations have been reported in the SSC of axotomised MNs^45^. The size of the VAChT immunoreactive profiles present in the presynaptic compartment of afferent nerve terminals contacting axotomised MNs showed changes similar to those found for NRG1 (area of VAChT-delimitated C-bouton in μm^2^: control = 6.80 ± 0.20 [n = 184], 14 days post-axotomy = 3.08 ± 0.14 [n = 114], and 30 days post-axotomy = 7.45 ± 0.32 [n = 153]; control *vs.* 14 days post-axotomy, p < 0.001; control *vs.* 30 days post-axotomy, non-significant; Student’s *t*-test). By contrast, the number of VAChT positive terminals declined more markedly (>45%, 7 days after axotomy) than that of NRG1 clusters (number of VAChT positive profiles per 100 μm^2^: control = 3.19 ± 0.3 [n = 14], 7 days post-axotomy = 1.46 ± 0.16 [n = 10]; p < 0.001, Student’s *t*-test). We observed that during microglial recruitment and activation around axotomised MNs, C-bouton sites exerted an apparent chemoattractant-like effect on microglial profiles (Figs 8b,c and 9a–c). When the proportion of distinct types of presynaptic terminals contacted by microglial profiles was measured in MN somata 7 days after axotomy (Fig. 9d–g), we found that more than 50% of VAChT-positive or VGluT1-positive terminals were intimately associated with microglial processes, whereas a much lower proportion of microglial profiles were in contact with GABAergic or serotoninergic synapses (Fig. 9h); data concerning the relative density of the distinct types of afferents do not account for these differences (not shown). In addition to motor axons, peripheral nerve injury leads to the interruption of the sensory proprioceptive axons that establish monosynaptic VGluT1-positive afferent connections with MNs^46^. It would not be surprising if there were an association between microglia with degenerating and presumably chemoattractant VGluT1-containing terminals^47^. Nevertheless, the C-bouton spatial specificity as a target for microglial migration is less expected, because the interneurons, from which these terminals originate, are not directly damaged by axotomy. This apparent chemotactic activity is probably related to the complex arrangement of molecules and signal-transduction pathways inherent to C-boutons. This may represent an early event in the inflammatory response to injured MNs that could contribute to the generation of a perisynaptic environment that could play a role in determining MN survival or degeneration. Further work is necessary to determine the molecular cues that attract microglial processes to C-type synapses following nerve injury.

## Discussion

With the emergence of new data about the C-bouton structure and function, and its possible relevance to MN pathology, there has been considerable recent interest in this type of synapses^19^,^48^. Here, we extend our previous report showing that the NRG1-ErbB2/3 signalling module is expressed at C-boutons^18^, by providing a more detailed analysis of the compartmentation of these molecules and their relation with other proteins that are known to be specifically clustered at this synaptic site. The SSC, the ER-related organelle that anatomically defines C-boutons, is the site at which some of these proteins accumulate. This is the case of SR1, Kv2.1 and NRG1. In addition, we show that, within the SSC, these proteins reside in segregated regions that probably represent distinct, highly specific spatial domains. This may define spatially-restricted fields for different signal-transduction pathways in the post-synaptic compartment, which are specifically arranged in relation to presynaptic proteins. This would be consistent with the emerging concept of “trans-synaptic nanocolumns”^49^. One so far unsolved question that deserves further attention is whether SSC-resident proteins can translocate and/or interact with the post-synaptic membrane, as appears to be the case for Kv2.1 and SR1. Kv2.1 is a major component of delayed rectifier K^+^ channels which are involved in the modulation of neuronal excitability in various neuronal types^5^. In agreement with our findings, Kv2.1 channels have sometimes been identified in the most peripheral region of the SSC^38^,^39^. These data indicate that Kv2.1 clusters are strategically situated at specific somatic synapses in conjunction with other signalling proteins including S1R and NRG1. S1R is a highly dynamic chaperone-like protein present in the SSC, which is involved in ALS when mutated^50^. S1R may operate here in a similar way to that described for mitochondrion-associated ER membrane in Ca^2+^ signalling via IP3 receptors, perhaps in collaboration with M2-receptor cholinergic activation^50^,^51^,^52^. Thus, by modulating spatiotemporal calcium signals, S1Rs may regulate either voltage-gated calcium channels or potassium channels^53^, both of which have an impact on neuronal excitability through a mechanism that acts via either G-protein-dependent M2 receptor activation or channel phosphorylation. In addition, it should be taken into account that highly clustered Kv2.1 phosphorylated proteins may have non-conducting, as yet undefined, functions^54^. It has been suggested that these Kv2.1 clusters could be sites for the delivery of membrane proteins to the cell surface^55^. Accordingly, Kv2.1 platforms may play a role as an organizer of the complex molecular assembly at the C-bouton postsynaptic site, regulating site-directed vesicle-membrane trafficking. This would perhaps explain the particular, belt-like, distribution of Kv2.1 protein, surrounding the whole oval-shaped C-bouton site.

The existence of NRG1 within the SSC facing ErbBs in association with cholinergic terminals is a recently described observation^18^ that we have extended on here. It has also been recently shown that NRG2 accumulates in the SSCs of cortical interneurons expressing ErbB4^39^. In this case, NRG2/ErbB4 signalling appears to regulate NMDA receptors in an autocrine manner. Although our data indicate that the main pathway for NRG1-ErbBs signalling at C-boutons may be retrogradely directed from post- to pre-synaptic compartments, we cannot rule out the possibility of additional autocrine NRG1 mediated signalling in MNs, as ErbBs are also expressed in these cells. Although our results concerning the localization of ErbBs contrast with those reported by Lasiene *et al*.^35^ using non-phosphospecific anti-ErbBs antibodies, we are confident of our results since they were extensively replicated and analysed using several different antibodies. The strongest presynaptic ErbB signal was obtained when phosphospecific anti-ErbB2 and ErbB4 antibodies were used; this points to the activation of this pathway in a particular subset of C-boutons. Furthermore, the NRG1/ErbB signalling system is also involved in a variety of well-established roles within the neuromuscular system. For example the MN-derived NRG1 pathway is critical: (i) to the development of Schwann cells and myelination^56^, (ii) to the development and plasticity of neuromuscular junctions^57^ and, (iii) perhaps also, to the induction of local AChR synthesis^58^,^59^. The anti-NRG1 antibody used in this work recognises a C-terminal epitope which is common within the different NRG-1 isoforms. The above mentioned actions are mainly mediated by the type III NRG1 (NRG1-III), which is the main isoform expressed in MNs^58^,^60^,^61^,^62^,^63^. It is therefore likely that the isoform we detected in C-boutons corresponds to NRG1-III, which is characterised by a cysteine-rich domain (CRD) determining a second N-terminal transmembrane domain. Signalling mediated by this membrane-anchored isoform may take place in a contact-dependent juxtacrine manner, through either the full length or truncated protein. Paracrine signalling via the proteolytic liberation of the EGF-like domain has also been described^64^. How post-synaptic NRG1 at C-boutons gains access to presynaptic ErbB receptors is not yet understood. Intercellular vesicular transmission mediated by exosomes may have a role in some forms of trans-synaptic communication^65^. We have observed intersynaptic multivesicular bodies at C-boutons with exosome-like vesicles containing NRG1^18^. It should be noted that the intersynaptic trafficking of large vesicles has already been documented in C-type synaptic terminals^66^. Another aspect that should be taken into account is the possibility of ErbBs-elicited NRG1 back signalling after the proteolytic release of the intracellular domain of NRG1, which may act as a transcriptional factor capable of regulating neuronal survival^67^.

Surface-associated NRG1 spots appeared in developing MNs on E18, at the same time as VAChT-positive perisomatic puncta. This is consistent with reported data on the development of cholinergic terminals in MNs^68^. However, the transient existence of large and un-innervated NRG1 spots in immature MNs suggests that SSC development is a cell autonomous process, which undergoes remodelling induced by nerve terminals during cholinergic synaptogenesis. In adults, SSC markers, including NRG1, are progressively lost after peripheral nerve section, together with microglial activation and synaptic loss. This is consistent with the decrease in NRG1-III expression found in the facial nucleus after axotomy^14^ and also with the redistribution of C-bouton-associated Kv2.1 channels after peripheral nerve injury^22^.

Another interesting question that has derived from our study is whether C-bouton has a particular role in the orchestration of a neuroinflammatory response. Our results suggest that C-bouton associated molecules act as cues for the attraction of microglial processes which, in turn, remove the synaptic terminals. In addition to motor axons, peripheral nerve injury leads to the interruption of the sensory proprioceptive axons that establish monosynaptic VGluT1-positive afferent connections with MNs^46^. It would not be surprising if there were an association between microglia with degenerating and presumably chemoattractant VGluT1-containing terminals^47^. Nevertheless, the C-bouton spatial specificity as a target for microglial migration is less expected, because the interneurons, from which these terminals originate, are not directly damaged by axotomy. This apparent chemotactic activity is probably related to the complex arrangement of molecules and signal-transduction pathways inherent to C-boutons. At the molecular level, one candidate for this action could be the NRG1/ErbBs signalling pathway. It has been shown that ErbB receptors are expressed in microglia and that NRG1 is a chemotactic factor for microglia *in vitro;* the NRG1/ErbBs module is specifically activated in spinal cord dorsal horn during microglial reaction following peripheral nerve injury^69^. This may represent an early event in the inflammatory response to injured MNs that could contribute to the generation of a perisynaptic environment that could play a role in determining MN survival or degeneration. However, we did not observe ErbB positive immunostaining in activated microglia around axotomised MN somata (data not shown).

Our findings reveal the highly specific arrangement of C-bouton-associated proteins, their disruption in acutely lesioned MNs and their putative role in regulating the neuroinflammatory response, and emphasize the relevance of this hitherto poorly understood structure in the physiology and pathology of MNs. The significance of NRG1-ErbBs retrograde signalling in C-type synapse deserves particular attention in the future as a putative new target for therapy in ALS.

## Material and Methods

### Animals, surgical procedures and tissue preparation

CD1 mice were purchased from Harlan Laboratories (Castellar del Vallès, Barcelona, Catalonia, Spain). All the animal experimentation procedures were performed according to the European Committee Council Directive and the norms established by the *Generalitat de Catalunya* (published as a law in the *Diari Oficial de la Generalitat de Catalunya* [DOGC] 2073, 1995). All the experiments were previously evaluated and approved by the Committee for Animal Care and Use of the University of Lleida.

For axotomy experiments in adult mice (postnatal day 60), the sciatic nerve was transected at the femoral level and its proximal stump was ligated in order to prevent spontaneous reinnervation.

To retrograde label MNs innervating the tibialis anterior (TA) and soleus muscles, cholera toxin B conjugated with either AlexaFluor 555 (for TA) or AlexaFluor 488 (for soleus) (1 μg/μl in phosphate buffer [PB], Molecular Probes, Eugene, OR, United States) was used. After surgical exposure of the muscles, volumes of 6 or 3 μl of CTB solution were respectively injected into the right TA or left soleus. One day after injection, the animals were fixed by transcardial perfusion, as described below.

All surgical manipulations were performed under anaesthesia, with a combination of ketamine (100 mg/Kg) and xylazine (10 mg/Kg). To minimise suffering, the animals were subjected to postoperative analgesia with intraperitoneally injected buprenorphine (0.05 mg/Kg).

### Spinal cord cultures

Primary cultures of dissociated spinal cord from CD1 mouse embryos (embryonic day 13) were prepared as previously described^70^ with minor modifications. Briefly, the lumbar spinal cords were dissected and the meninges and ganglia removed. Dissociated cells were plated at a density of 300,000 per well in 12-well Nunclon culture dishes containing round glass coverslips coated with a poly-D-lysine plus Matrigel basement membrane matrix (Corning, Bedford, MA). The cells were then maintained in minimum essential medium (Gibco, Waltham, USA) enriched with 5 g/l glucose and supplemented with 3% horse serum, 10 ng/ml nerve growth factor and B27 medium (Gibco). On day 6, the cultures were treated with 1.4 μg/ml cytosine-β-arabinoside (Sigma-Aldrich, Saint Louis, MO) in order to minimise the growth of non-neuronal cells. Cultures kept for 14–28 days *in vitro* were washed in PBS, fixed in 4% paraformaldehyde (PFA) in 0.1 M PB (pH 7.4) for 1 h, and processed for immunofluorescence.

### Multiple fluorescent labelling and confocal microscopy

Tissue samples were obtained from mice transcardially perfused with 4% PFA in 0.1 M PB, pH 7.4. Lumbar spinal cord samples were dissected, post-fixed in the same fixative overnight at 4 °C, and then cryoprotected with 30% sucrose in 0.1 M PB containing 0.02% sodium azide. Transverse cryostat sections (16-μm thick) were collected on gelatin-coated glass slides.

Sections were then permeabilised with phosphate-buffered saline (PBS) containing 0.1% Triton X-100 for 1 h, blocked with either 10% normal goat serum or normal horse serum in PBS for 1 h at room temperature, and then incubated overnight at 4 °C with an appropriate primary antibody mixture. The primary antibodies used are indicated in Table 1.

Once previously washed with PBS, sections were incubated for 1 h with a combination of appropriate secondary fluorescent antibodies labelled with one of the following fluorochromes (1/500): Alexa Fluor 488, Alexa Fluor 546, (Molecular Probes, Eugene, OR, United States), Cy3, or Cy5 (Jackson Immuno Research Laboratories, West Grove, PA, United States). Finally, the spinal cord sections were labelled with blue fluorescent NeuroTrace Nissl staining (1:150; Molecular Probes) and mounted using an anti-fading medium containing 0.1 M Tris-HCl buffer (pH 8.5), 20% glycerol, 10% Moviol, and 0.1% 1,4-diazabicyclo[2,2,2]octane. For ErbB visualisation, some sections were processed using the tyramide signal amplification (TSA), following the procedure recommended by the manufacturer (ThermoFisher, Waltham, MA).

For the 3D analysis of C-boutons in individual MNs, a squash procedure^71^ was used. Fixed spinal cords were sectioned with a vibratome (200-μm thick). Sections were collected in PB, and placed on SuperfrostPlus microscope slides (Menzel-Glaser, Germany). A coverslip was then applied to the tissue which was squashed with forceps. The preparations were then frozen in liquid N_2_, and, after removing the coverslip, the slides with the retained tissue were fixed in methanol at −20 °C for 10 min. After washing with PBS, immunocytochemistry was performed as described above.

The slides were then examined under a FluoView FV-500 or FluoView FV-1000 Olympus laser-scanning confocal microscopes (Olympus, Hamburg, Germany). The MNs were imaged after obtaining optical sections (0.5 or 1 μm) of cell bodies. Digital images were analysed with either Visilog 6.3 software (Noesis, Orsay, France) or ImageJ software (US National Institutes of Health, Bethesda, MD, USA). For colocalization analysis the ImageJ plugin developed by Pierre Bourdoncle (bourdoncle@ijm.jussieu.fr) was used.

Immunolabelled profiles of NRG1 and of the different protein markers examined were then manually counted on the screen for each MN soma. The area and perimeter of MN somata, and microglial profiles covering the MNs were also manually measured. The number of synaptic boutons contacting activated microglia in axotomised MNs was evaluated by image analysis (ImageJ). After application of the outline tool on binarised IBA1 images, these were merged with those corresponding to binarised synaptic boutons: the number of boutons contacting perisomatic microglial profiles was manually counted. Three-dimensional reconstructions were performed using Bitplane (Imaris, Bitplane, CT, USA) on 0.5-μm thick Z step obtained with the confocal microscope. The digital images were edited using FV10-ASW 3.1 Viewer (Olympus) and Adobe Photoshop CS4 (Adobe Systems Inc, San Jose, CA).

### Electron microscopy

Some of the animals were perfused either with1% PFA and 1% glutaraldehyde in 0.1 M PB (pH 7.4) for conventional electron microscopy or with 4% PFA and 0.2% glutaraldehyde in 0.1 M PB for immunoelectron microscopy.

For conventional electron microscopy, dissected tissues were postfixed in 1% OsO_4_ and processed for Embed 812 embedding according to standard procedures. Ultrathin sections were counterstained with uranyl acetate and lead citrate.

For ultrastructural immunolabelling of NRG1, either pre-embedding or post-embedding (freeze substitution and low temperature embedding in Lowicryl HM20 resin [Electron Microscopy Sciences, Hatfield, PA, USA]) procedures were used, as previously described^18^. Double immunolabelling of NRG1 in combination with SR1 or Kv2.1 was performed in ultrathin cryosections obtained with a Leica EM FC cryoultramicrotome and processed according to established methods^72^,^73^. To facilitate the localisation of C-boutons in ultrathin cryosections, hypoglossal, instead of spinal cord, MNs were analysed in some cases. To do this, samples from brainstem or spinal cord were sectioned with a vibratome (200-μm thick), and regions containing either hypoglossal nuclei or ventral horn were microdissected. Ultrathin cryosections were labelled using rabbit anti-NRG1 antibody (1:50; Santa Cruz Biotechnology, sc-348) in combination with either mouse anti-Kv2.1 (1:30; NeuroMab, 73014) or mouse anti-S1R (1:10; Santa Cruz Biotechnology, sc-137075). After washing, samples were incubated with 12 nm gold-conjugated goat anti-rabbit IgG (1:30; Sigma Aldrich) and 5 nm gold-conjugated goat anti-mouse IgG (1:30; Sigma Aldrich). Controls, omitting the primary antibody, were also performed. Observations were performed with either a Jeol JEM 1400 (Jeol, Tokyo, Japan) or a Zeiss EM 910 (Zeiss, Jena, Germany) transmission electron microscope. The subcellular distribution of immunoglod labelling of NRG1 in MNs was evaluated by the stereological analysis described by Mayhew^37^.

### Statistical analysis

The data are expressed as means ± SEM. The statistical analysis was assessed by either the Student’s *t-*test or one-way analysis of variance (ANOVA) followed by *post hoc* Bonferroni’s test. Chi-square was used for the stereological analysis of immunogold labelling. The level of significance was established at *p* < 0.05.

## Additional Information

**How to cite this article**: Casanovas, A. *et al*. Neuregulin 1-ErbB module in C-bouton synapses on somatic motor neurons: molecular compartmentation and response to peripheral nerve injury. *Sci. Rep.* **7**, 40155; doi: 10.1038/srep40155 (2017).

**Publisher's note:** Springer Nature remains neutral with regard to jurisdictional claims in published maps and institutional affiliations.

## Supplementary Material

Supplementary Information

## Figures and Tables

**Table 1:** Antibodies used for immunocytochemistry.

Target	Source	Host species	Used concentration
Choline acetyltransferase (ChAT)	Millipore (Tamecula, CA) (AB 143)	Rabbit polyclonal	1:200
Glial fibrillary acidic protein (GFAP)	Abcam (ab4674)	Chicken polyclonal	1:1000
Her4/ErbB4	Cell Signalling (#4795)	Rabbit monoclonal	1:50
Ionised calcium-binding adaptor molecule 1 (IBA1)	Abcam (ab5076)	Goat polyclonal	1:500
Kv 2.1 voltage-gated potassium channel (Kv2.1)	NeuroMab (73–014)	Mouse monoclonal	1:100
M2 muscarinic receptor	Alomone Labs (AMR-002)	Rabbit polyclonal	1:100
Neu (C-18) (ErbB2)	Santa Cruz Biotechnology (Sc-284)	Rabbit polyclonal	1:50
NRG1 1α/β1/2	Santa Cruz (sc-348)	Rabbit polyclonal	1:200
Phospho-Her4/ErbB4 tyr1248	Cell Signalling (#4757)	Rabbit monoclonal	1:50
p-Neu tyr1248 (p-ErbB2)	Santa Cruz (sc-293110-R)	Rabbit polyclonal	1:100
Sigma-1 receptor (S1R)	Santa Cruz (sc-137075)	Mouse monoclonal	1:50
Serotonin	Chemicon (MAB352)	Rat monoclonal	1:100
SK3 calcium-activated potassium channel (SK3)	Alomone Labs (APC-025)	Rabbit polyclonal	1:100
SMI32	Covance Research Products Inc (SMI32)	Mouse monoclonal	1:5000
Synaptophysin	Synaptic Systems (101 004)	Guinea pig polyclonal	1:500
Vesicular acetylcholine transporter (VAChT)	Synaptic Systems (139 105)	Guinea pig polyclonal	1:500
Vesicular GABA transporter (VGAT)	Synaptic Systems (131 004)	Guinea pig polyclonal	1:200
Vesicular glutamate transporter 1 (VGluT1)	Synaptic Systems (135 304)	Guinea pig polyclonal	1:500
Vesicular glutamate transporter 2 (VGLuT2)	Synaptic Systems (135 404)	Guinea pig polyclonal	1:500
