# Supplementary material for: Neuregulin 1-ErbB module in C-bouton synapses on somatic motor neurons: molecular compartmentation and response to peripheral nerve injury
Source: Sci Rep. 2017 Jan 9;7:40155. doi: 10.1038/srep40155 (PMC5220293; doi:10.1038/srep40155)
Supplement: Supplementary Information [file srep40155-s1.pdf]

Supplementary Information

Neuregulin 1-ErbB module in C-bouton synapses on somatic motor neurons: molecular compartmentation and response to peripheral nerve injury

Anna Casanovas, Sara Salvany, Víctor Lahoz, Olga Tarabal, Lídia Piedrafita, Raimundo Sabater, Sara Hernández, Jordi Calderó & Josep E. Esquerda*

Supplementary Figure 1. Ultrastructure of C-type synapses at the surface of MN soma. (**a-d**) Synaptic compartments were pseudo-coloured: presynaptic terminals, green; MN soma (postsynaptic), red; subsynaptic cistern (SSC), and ER, blue; intersynaptic space, yellow. Presynaptic terminals show some mitochondria and synaptic vesicles; in the postsynaptic compartment, the ER-related SSC is seen closely adjacent to postsynaptic membrane (arrows in **a**, **b** and **c**). The C-bouton in (**a**) was taken from an adult mouse whereas in (**b**) and (**c**) it came from a newborn mouse. A detail of the organisation of the compartments at the C-bouton synapse is depicted in (**d**): 1 = presynaptic, 2 = intersynaptic extracellular space, 3 = postsynaptic cytoplasmic compartment lodged between the postsynaptic membrane and the SSC, 4 = SSC, and 5 = MN cytoplasm. Scale bars: in **(a**, **b**, and **c)** = 250 nm; in **(d)** = 40 nm.

Supplementary Figure 2. Immunostaining of NRG1 receptors ErbBs (green) in conjunction with VAChT (red) demonstrates the presynaptic localisation of ErbBs. (a-c) ErbB2 immunoreactivity after applying the tyramide signal amplification (TSA) procedure shows positive signal in MN somata and neuropil, without association with VAChT positive C-boutons. **(d-f)** By using a phosphospecific anti-ErbB2 antibody a faint signal is detected in association with C-boutons (arrows). **(g-i)** ErbB3 immunoreactivity is low in MNs, without any trace of

positive immunoreactivity in association with presynaptic VAChT. **(j-o)** Both unphosphorylated **(j-l)** and phosphorylated **(m-o)** ErbB4 immunoreactivity can be detected in association with some VAChT positive C-boutons (arrows) **(p-r)** ErbB4 immunoreactivity unambiguously colocalises with VAChT (arrows) after using the TSA procedure. **(s-u)** An enlarged C-bouton showing the colocalisation of ErbB4 **(s)** and VAChT **(t)**. Scale bars: in **(r)** = 20 µm (also valid for **(a-q)**); in **(u)** = 2 µm (valid for **(s,t)**).

Supplementary Figure 3. Fast and slow MNs display differential morphometrical parameters on C-bouton-associated NRG1. (a,b) Fast **(a)** and slow **(b)** MNs were identified after cholera toxin B (red) retrograde tracing following its injection into the tibialis anterior(TA) or soleus muscles, respectively. NRG1 (green) immunolabelling was analysed in both MN populations. **(c,d)** Graphs showing the density **(c)** and size **(d)** of C-boutons containing NRG1 on TA and soleus MNs. The data are expressed as mean ± SEM of n=15-22 3D reconstructed MNs **(c)** and n=199-322 spots **(d)**. ***p< 0.001 (Student's t-test). Scale bar: in **(b)** = 20 µm (valid for **(a)**).

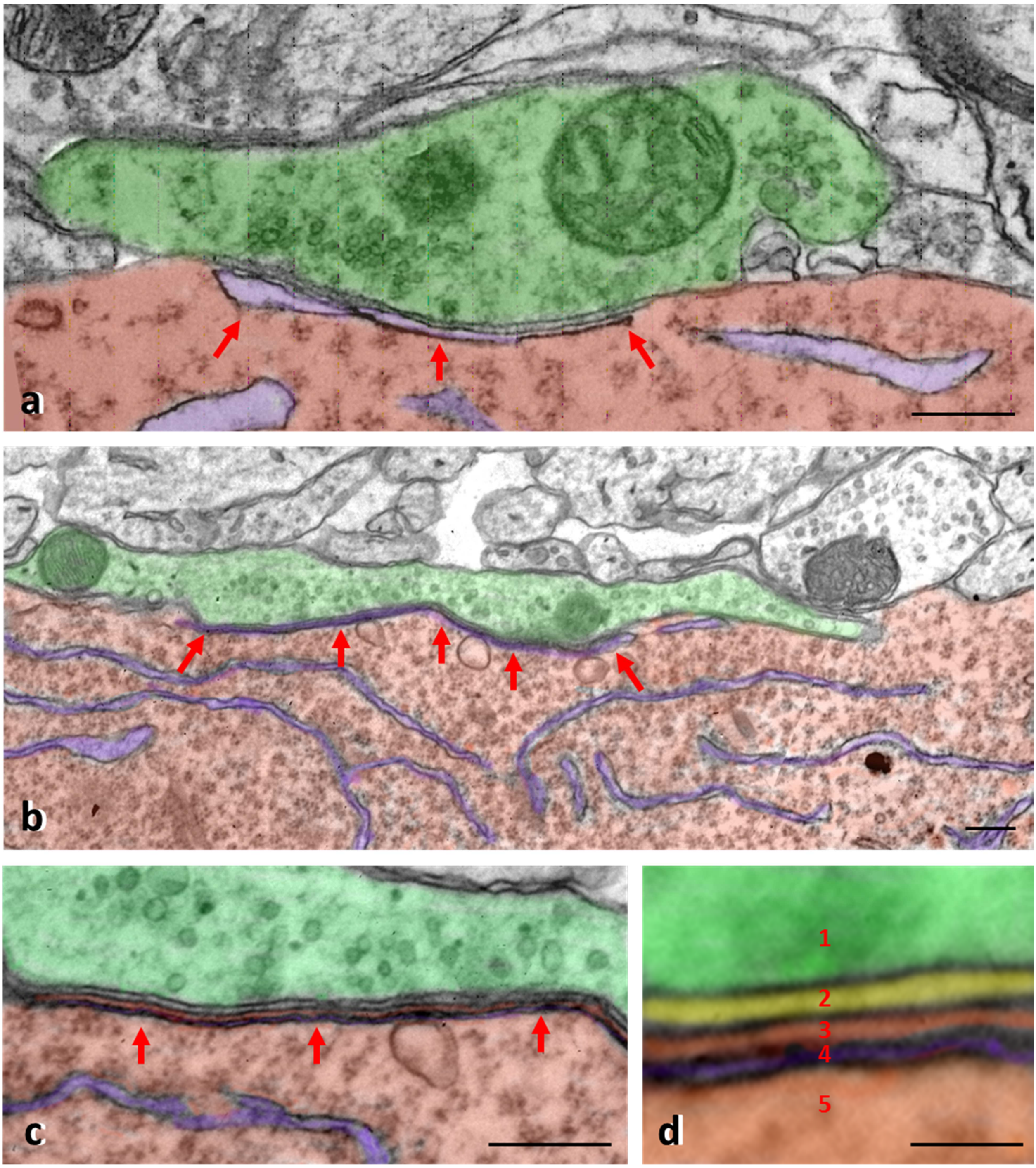

Supplementary Fig. 1

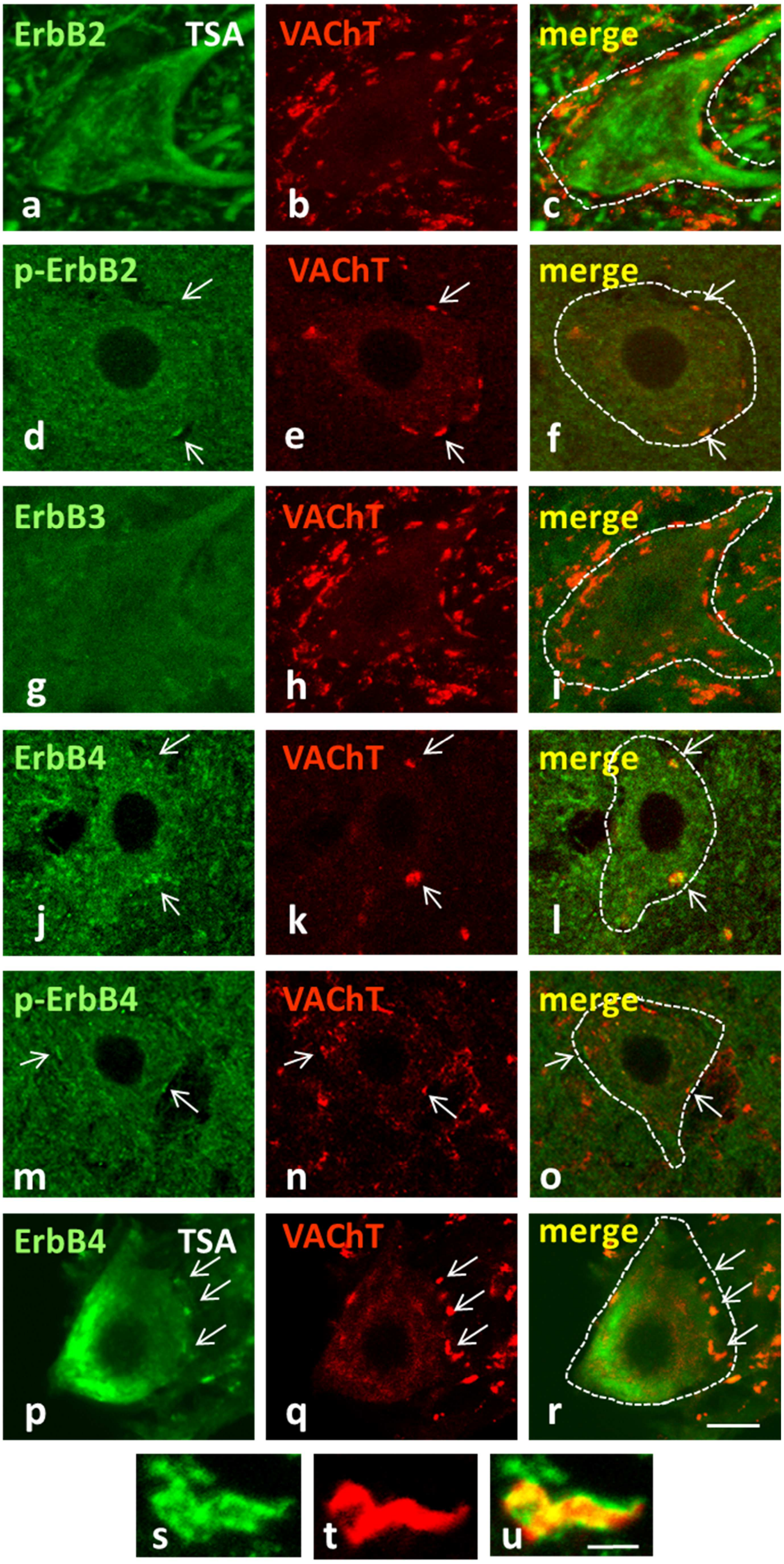

Supplementary Fig. 2

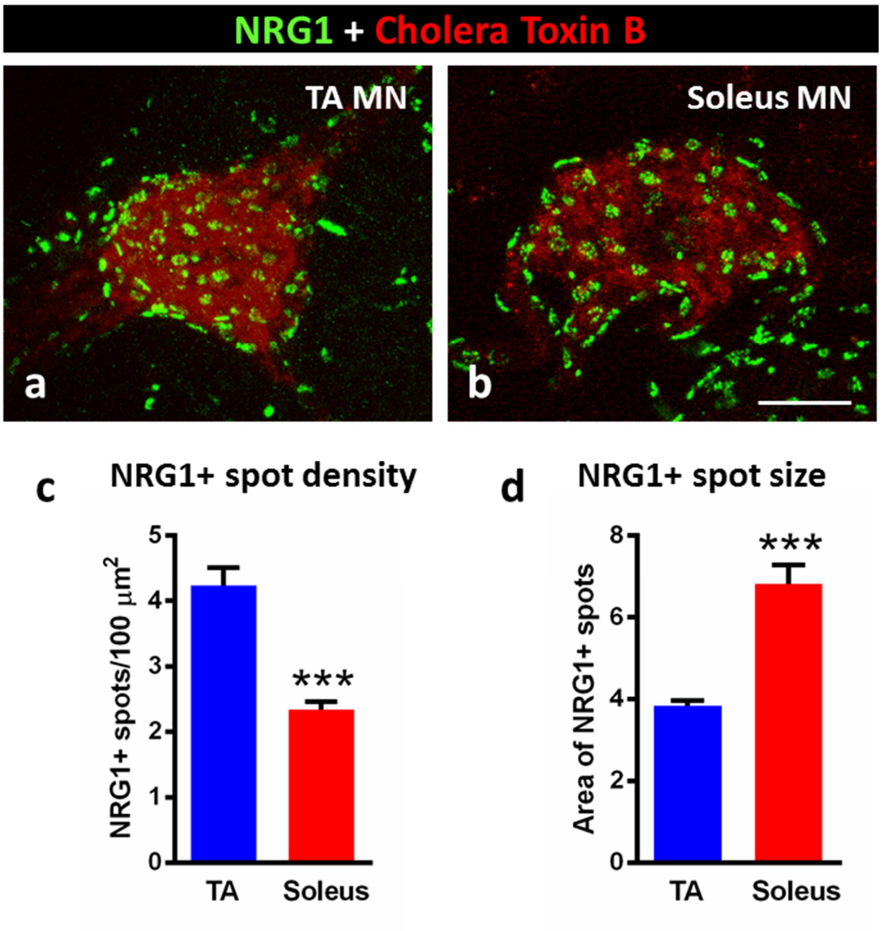

Supplementary Fig. 3
